# Pharmacists’ clinical roles and activities in inpatient hospice and palliative care: a scoping review

**DOI:** 10.1007/s11096-023-01535-7

**Published:** 2023-02-11

**Authors:** Ursina Wernli, Désirée Hischier, Christoph R. Meier, Sibylle Jean-Petit-Matile, Andrea Kobleder, Carla Meyer-Massetti

**Affiliations:** 1 Clinical Pharmacology and Toxicology, Inselspital University Hospital Bern, Bern, Switzerland; 2 Graduate School for Health Sciences (GHS), University of Bern, Bern, Switzerland; 3 Clinical Pharmacy and Pharmacoepidemiology, University of Basel, Basel, Switzerland; 4 Hospice of Central Switzerland, Lucerne, Switzerland; 5 Institute of Applied Nursing Science, Eastern Switzerland University of Applied Sciences OST, St. Gallen, Switzerland

**Keywords:** Hospice and palliative care, Medication reconciliation, Medication review, Medication safety, Pharmacist, Pharmacy services

## Abstract

**Background:**

Pharmacists contribute to medication safety by providing their services in various settings. However, standardized definitions of the role of pharmacists in hospice and palliative care (HPC) are lacking.

**Aim:**

The purpose of this scoping review was to provide an overview of the evidence on the role of pharmacists and to map clinical activities in inpatient HPC.

**Method:**

We performed a scoping review according to the PRISMA-ScR extension in CINAHL, Embase, and PubMed. We used the American Society of Hospital Pharmacists (ASHP) Guidelines on the Pharmacist’s Role in Palliative and Hospice Care as a framework for standardized categorization of the identified roles and clinical activities.

**Results:**

After screening 635 records (published after January 1st, 2000), the scoping review yielded 23 publications reporting various pharmacy services in HPC. The articles addressed the five main categories in the following descending order: ‘*Medication order review and reconciliation*’, ‘*Medication counseling, education and training*’, ‘*Administrative Roles*’, ‘*Direct patient care*’, and ‘*Education and scholarship*’. A total of 172 entries were mapped to the subcategories that were added to the main categories.

**Conclusion:**

This scoping review identified a variety of pharmacists’ roles and clinical activities. The gathered evidence will help to establish and define the role of pharmacists in inpatient hospice and palliative care.

**Supplementary Information:**

The online version contains supplementary material available at 10.1007/s11096-023-01535-7.

## Impact statements

- A variety of clinical pharmacy activities were identified to help establish a standardized definition of pharmacists’ roles in inpatient HPC.
- Among the identified clinical activities, pharmacist-led systematic medication reviews and drug therapy adjustments to optimize medication regimens were the most commonly reported.

## Introduction

In hospice and palliative care (HPC), drug therapy is focused on decreasing patients’ symptom burden and improving their quality of life [1]. However, end-of-life medication has to balance complex factors [2]. On average, palliative care patients receive 7.0–7.8 drugs daily [2–4]. Drug-related problems (DRPs), encompassing mainly inadequate drug treatment, inappropriate dosages, drug–drug interactions, adverse drug events, medication errors, and poor adherence [5, 6], may arise from the patients’ general vulnerability, their comorbidities, and the high prevalence of polypharmacy [7, 8]. To prevent potential harm from DRPs, it is necessary to enable rational and appropriate prescribing, to decrease prescribing errors, and to identify potential DRPs [9, 10].

A 2021 German study in patients of a hospital-based palliative care (PC) unit demonstrated DRPs’ impact on symptom progression. As symptom control requirements and medication regimens became more complex, DRPs arose more frequently. Pharmacists’ medication reviews and subsequent recommendations for action led to successful detection and interventions to resolve the identified DRPs, while maintaining adequate symptom control [11].

The body of evidence demonstrating clinical and economic benefits of pharmacy services, encompassing pharmaceutical care [12] to increase medication safety in various settings is growing [13–15]. Defined as a subset of clinical pharmacy practice, pharmaceutical care contributes to the care of individuals [12]. According to the American Society of Hospital Pharmacists, pharmaceutical care is the “responsible provision of medication-related care for the purpose of achieving definite outcomes that improve a patient’s quality of life” [16] which is in accordance with the main principle of HPC to maximize quality of life wherever possible [17, 18]. Although there have been major advances in defining the role of pharmacists in HPC [19, 20], standardization is lacking. This was revealed in a 2021 qualitative study on the role of hospice pharmacists in the UK, a country with advanced pharmacy services in various settings [21]. There is a lack of a standardized definition not only in Europe but also on a national level. In Switzerland, the role of pharmacists has not yet been defined and a recent nationwide study on PC networks failed to include pharmacists [22]. Occasionally, clinical pharmacists perform medication reviews and join rounds in hospital-based PC units, as they do in other medical specialties [23]. To our knowledge, only one Swiss hospice or hospice-like institution (i.e., hospitals excluded) collaborates with a clinical pharmacist on a contractual basis (local survey performed in 2021) [24].

A European whitepaper on standards and norms for HPC lists pharmacists as members of the multiprofessional team in the provision of PC services, yet fails to define their roles [25]. The COVID-19 situation contributed efforts to further develop the role of pharmacists in HPC [26] and pharmacy services in patients with advanced cancer are increasingly implemented [27]. However, the scope of HPC is much broader. Implementation of pharmacy services and the impact on clinical outcomes need further investigation.

In order to establish a definition of the role of pharmacists in HPC and to implement pharmacy services, an overview of the current evidence on the role of pharmacists and clinical activities in HPC is essential.

### Aim

The purpose of this scoping review was to provide an overview of the evidence of the role of pharmacists and to map clinical activities in inpatient HPC.

## Method

We performed and reported this scoping review according to the recommendation of the PRISMA extension for scoping reviews [28]. A protocol, that was not previously published, was used to outline the search strategy and to document the process.

### Search and information sources

We used the aspects location (‘L’), professionals (‘P’), and services (‘SE’) from the mnemonic ECLIPSE [29] to develop the search string, define the eligibility criteria, and structure the data charting process. The search string was designed combining the three building blocks ‘Hospice-like settings’, ‘Pharmacist’, ‘Pharmacy Services’, and ‘using MeSH terms and keywords. It was initially developed in PubMed and translated for use in Embase (using Emtree terms) and CINAHL (using Subject Headings) facilitated through the polyglot website [30]. The full electronic search strategy is available as a Supplementary file S1). Because provision of clinical pharmacy services is rapidly changing and adapting to the current needs [14], the search was limited to articles published after turn of the millennium (January 1st, 2000). The final search was performed on February 10th, 2021. No filters were applied. Employees of the university’s library were contacted for procurement of articles where full text versions were not available online. A weekly literature alert was set to identify relevant newly published articles. The latest alert check and hand-search to identify eligible articles that were published since February 2021 took place on October 10, 2022.

Although modern definitions stress that PC is a “component of comprehensive care throughout the life course” [31], this scoping review focuses on inpatient HPC settings as the ASHP guidelines used for mapping of the identified clinical pharmacy activities were developed from a hospital perspective (see “Synthesis of results” section).

### Eligibility criteria and selection of sources of evidence

After removing all duplicates, title-abstract screening was performed in Mendeley® by two independent reviewers (DH, UW) based on predefined eligibility criteria (see Table 1). Discrepancies were resolved through consensus. Full text screening was performed by one reviewer (DH) and discussed with two additional reviewers (UW, CMM).

**Table 1:** Eligibility criteria of the scoping review

	Inclusion	Exclusion
**C**lient group (E**C**LIPSE)	Patients (≥ 18 years old), with incurable illness and a life-expectancy ≤ 6 months^a^	Pediatric patients
**P**rofessionals (ECLI**P**SE)	Pharmacists	–
**L**ocation, **Se**rvice (EC**L**IP**SE**)	Inpatient hospice settings where pharmacy services are provided	Euthanasia, assisted suicide
Type and year of publication	Original work, practice reports, position papers, and guidelines published after January 1st of 2000	Commentaries, conference abstracts/presentations, editorials, interviews, reviews; published before 2000
Language	English, German, Spanish, Italian or French	Other languages
Accessibility	Abstract and full text fully accessible	No access to abstract and/or full text

^a^Rule of thumb of observed life-expectancy in hospice care (e.g., an indicator could be end-of-life care)

### Data charting process and data items

A table to collect charted data (author, country, year of publication, type of publication, study design, methods, setting, role of pharmacist, and clinical pharmacy activities) was created in Excel® with one row for each included article. Frequency of the mapped clinical activities, and, where applicable, their impact on clinical outcomes and findings from cost analyses were assessed. The data charting process of the included articles was discussed among the authors. The detailed table is provided by the authors upon request.

To standardize the heterogeneous definitions of the publication types included, the terms *practice research report* and *practice report* were applied to articles that deviated from the classical structure of an original article. Those reporting research performed in practice (e.g., qualitative research for implementation projects), but with no clear publication structure, were classed as *practice research reports*; narrative reports from practice sites were classified as *practice reports* (e.g., project progress reports, case studies).

### Synthesis of results

We created a table (see “Summary of identified pharmacy services provided to inpatient hospice and palliative care”, Supplementary file 2) to summarize the charted data. We categorized the identified clinical activities and mapped them to the following five main categories of the American Society of Hospital Pharmacists (ASHP) Guidelines on the Pharmacist’s Role in Palliative and Hospice Care [19]:

- ‘*Direct patient care*’: to serve as a resource on the optimal use of medication in symptom management (optimized outcomes), to optimize a medication regimen, and to improve adherence to a medication regimen.
- ‘*Medication order review and reconciliation*’: to manage and improve the medication-use process in patient care settings, to optimize a medication regimen and to increase patient safety as well as pharmacoeconomy.
- ‘*Medication counseling, education and training*’: to provide medication counseling, to train and to educate staff, patients, caregivers, and families.
- ‘*Administrative roles*’: to develop procedures that ensure safe use of medications, to optimize patient care services, to support medication supply chain management.
- ‘*Education and scholarship*’: to contribute to the body of knowledge of pharmacists in PC and further develop the role of pharmacists.

With these guidelines, the ASHP established a definition of the pharmacist’s role in HPC and provided a summary of general best practice principles. Further, the article was used to determine subcategories for each of the five main categories.

Findings of articles that investigated impact on clinical outcomes and/or performed a cost analysis of the pharmacy services (see “Summary of identified pharmacy services provided to inpatient hospice and palliative care”, Supplementary file 2) were separately mapped (see “Impact on clinical outcomes and findings from cost analyses”, Table 2).

**Table 2:** Impact on clinical outcomes and findings from cost analyses

	Impact on clinical outcomes	Findings from cost analyses
Atayee et al. [32]	Significantly improved length of stay, length from admission to palliative care consult, and time from consult to discharge or death in patients seen by palliative care pharmacist within 72 h; consolidated medication regimen with improved adherence; identification, avoiding and/or correction of potential harm from drug interaction/harmful doses and aberrant drug-taking behavior; coordinated discharge documents	
Basri et al. [33]	Physician suggested that time spent by pharmacist to provide direct patient care and to make medication-related interventions for hospice patients results in time freed up for other prescribers on the team, potentially enhancing their ability to perform patient care in more patients and administrative responsibilities	Estimated annual unadjusted cost avoidance of $1,417,602.90 (all interventions included), annualized adjusted cost avoidance total of $99,232.20 (based on conservative estimate that 7% of the interventions would be expected to prevent harm)
Hanley et al. [26]	Shorter length of stay compared to patients without pharmacist involvement (8.75 vs. 10 days), greater difference in patients discharged from hospital (7.9 vs. 11.8 days)	Cost avoidance (ScHARR Model) with estimated total cost avoidance of 61,824 GBP/month, which equals 38,287 GBP per 1 FTE monthly
Kaldy et al. [45]	*Stated by interviewees:* huge impact by contribution of expertise to helping patients with pain relief and enabling them to pass away peacefully, which is appreciated by nurses, physicians, and patients	
Kemp et al. [34]	Identification of discrepancies (i.e., errors of omission) at transition of care	
Knowlton et al. [49]	Doctors were able to save 36 min per hospice patient per week that would otherwise have been spent on pharmacy issues (time savings come from evidence-based therapies that are beneficial to the patient with patients getting the right medications and the families getting a better understanding of the process)	
Latuga et al. [35]		No significant associations between time spent by the pharmacist performing clinical, administrative, or dispensing services and the number of medication requirements and per diem medication costs; there was no significant difference between hospices that only have a pharmacist on staff, only use a PBM or those using a combination of a pharmacist and a PBM
Le et al. [36]	≥ 50% reduction in medications recommended for deprescribing (every 1-unit increase in the number of patient encounters with hospice pharmacists was associated with 3.2-fold higher odds of achieving a ≥ 50% reduction in medications that were recommended for deprescribing)	
Lehn et al. [37]	Contribution to the quality and value of care provided to a palliative care patient and their family, in a way that is left unsatisfied if this discipline’s perspective is not included in the care equation; physician could see an increased number of patients when the pharmacist is complementary to the palliative care team	Cost of a potential adverse drug event estimated between $5,900 (1993) and $15,340 per occurrence (2015), which equated to $1,076,018 (1993) or to $2,797,647 (2015) for a total annualized return on investment of 1.2 to 2.9 million $ for a palliative care pharmacist position with an average clinical pharmacist’s salary in the United States reported to range from $115,000–$124,000 (2018)
Malotte et al. [38]	Decrease in length of stay of 12.3 days in patients with pharmacist involved, significant difference in pain severity goal attainment (demonstrated of improvement shorter in comparison to other settings without pharmacist involved) and overall improved patient symptoms	
Pawlowska et al. [39]	*Emerged from survey*: proper storage of drugs, decreased costs of the therapy, and improved access to drugs but participants still do not acknowledge the clinical role of pharmacists; presence of a pharmacist can decrease the costs associated with therapy (but no cost analysis was performed)	
Quinn et al. [48]	*Author conclusion:* pharmacist's interventions will result in improved patient outcomes and reduction of costs associated with untoward effects	
Richter et al. [51]	Clinical impacts of expertise (reduction of unnecessary medications, improved assurance medication list continuously accurate and strategically focused formulary adherence, bowel regimens with opioid pain medications, elimination of medications with increased risk for falls) provided to interdisciplinary group by clinical pharmacist well regarded by physicians and nurses (regularly expression of high valuation) and appreciated as a positive impact on the quality and efficiency of patient care	Substantial financial impacts were made in the first year and PPD drug cost savings was substantial after only the first month and continued to improve over the following months: average PPD drug cost decreased from $5.44 (2016) to $4.07 (2017) and cost savings of $427,705 was observed (with an estimated $15,750 annually cost savings from hospitalization prevented by clinical pharmacist interventions)
Romero et al. [40]	Pharmacist-led intervention directed to improve continuous subcutaneous infusion of opioids processes in an inpatient hospice increased timeliness of medication delivery	
Wilson et al. [42]	A desired clinical outcome is more commonly achieved when the pharmacist’s recommendation is accepted (86.4% when accepted, 25% when declined), this variable was also the strongest predictor for patients achieving a desired clinical outcome (OR, 19.0; 95% CI, 7.10–50.93; *P* < 0.001)	

*FTE* full time equivalent, *GBP* Great British Pounds, *PBM* pharmacy benefit manager, *PPD* per patient-day

## Results

### Selection of sources of evidence

**Fig. 1:**
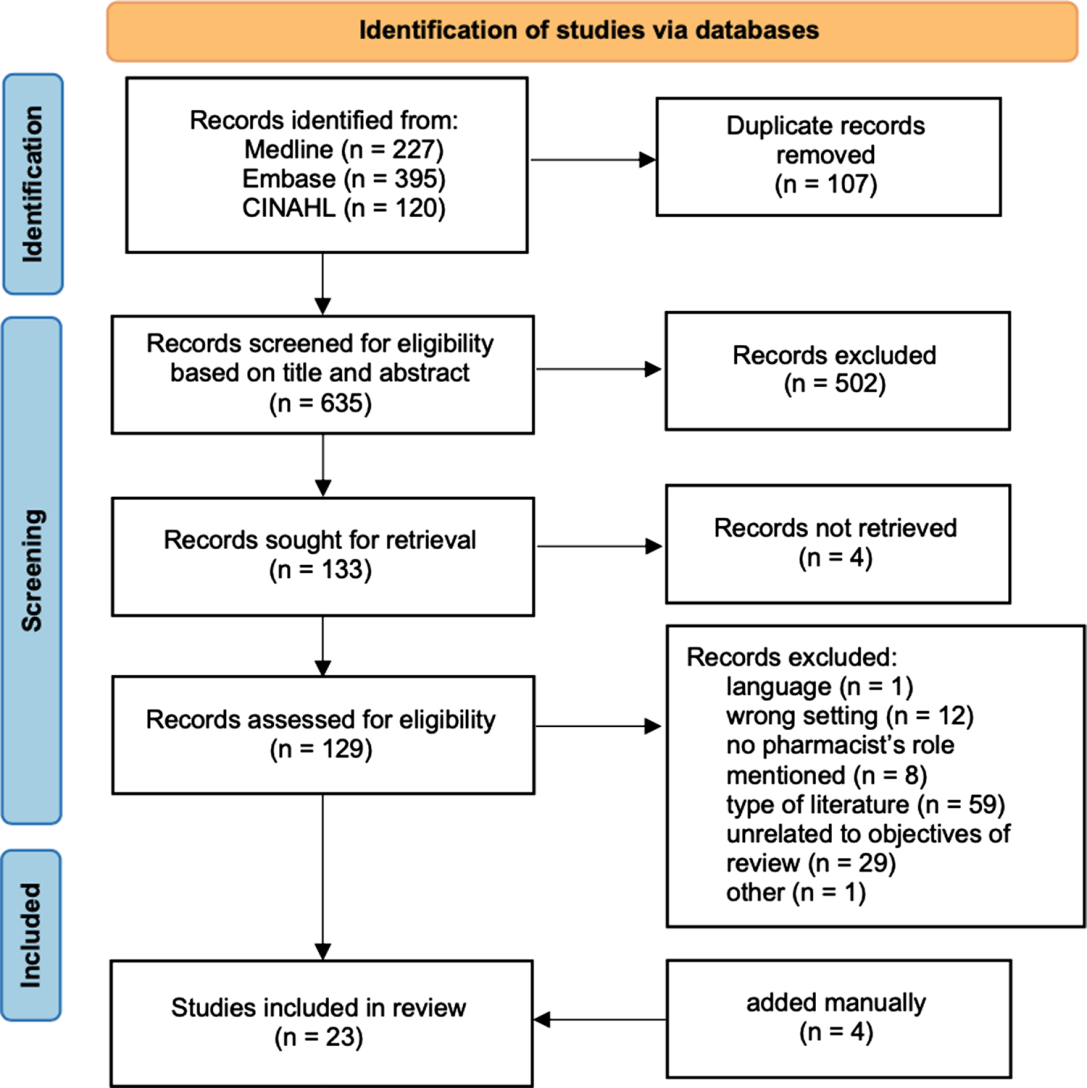
Flowchart of the scoping review [28]

A total of 742 publications were identified in the three databases. After removal of duplicates, 635 articles were screened for eligibility based on title and abstract. We assessed 129 articles for eligibility based on full text. The scoping review yielded a total of 23 publications, of which 22 were further used for data charting (see Fig. 1). Although identified in the literature search, the clinical roles and activities listed in the ASHP guidelines were not included in the data charting process. However, the article served as an indicator article to confirm that the search strategy had included the most relevant publications on the topic.

### Characteristics of sources of evidence

Of the included sources, 77.3% (n = 17/22) originated from the United States of America (USA). The others sources originated from the United Kingdom (n = 3/22, 13.6%), Poland (n = 1/22, 4.5%), and Qatar/Canada (n = 1/23, 4.5%). The majority of the included articles (n = 12/22, 54.5%) were original articles [21, 32–42], followed by articles classified as practice reports (n = 6/22, 27.3%) [43–48] and practice research reports (n = 4/22, 18.2%) [26, 49–51].

### Synthesis of results

Table 2 in Supplementary material shows a summary of all pharmacists’ roles and clinical activities. Overall, the articles revealed a wide range of pharmacists’ clinical roles and activities as well as pharmacy services provided to inpatient HPC settings (see “Summary of identified pharmacy services provided to inpatient hospice and palliative care”, Supplementary file 2).

The frequency of the five main categories mentioned among the 22 articles was assessed with the following descending order observed: ‘*Medication order review and reconciliation*’ (n = 20/22, 90.9%), ‘*Medication counseling, education and training*’ (n = 19/22, 86.4%), ‘*Administrative Roles*’ and ‘*Direct Patient Care*’, both (n = 16/22, 72.7%), and ‘*Education and Scholarship*’ (n = 10/22, 45.5%).

A total number of 172 entries (N = 100%) was mapped to the five main categories in descending order of frequency: ‘*Medication Counseling, Education and Training*’ (n = 46/172, 26.7%), ‘*Administrative Roles*’ (n = 44/172, 25.6%), ‘*Direct Patient Care*’ (n = 39/172, 22.7%), ‘*Medication Order Review and Reconciliation*’ (n = 26/172, 15.1%), and ‘*Education and Scholarship*’ (n = 17/172, 9.9%).

Subcategories were added to each main category to summarize and assess the identified clinical activities more accurately. Most of the articles addressed the subcategory ‘*Medication review (optimizing medication regimens and drug therapy adjustments)’* (n = 20/22, 90.9%), followed by ‘*Medication counseling, training, and education to health care providers’* (n = 17/22, 77.3%) and ‘*Medication counseling, training, and education to patients, caregivers, and families’* (n = 15/22, 68.2%).

The same three subcategories represented the most entries of clinical activities.

### Results of individual sources of evidence

As both, the subcategory ‘*medication review (optimizing medication regimens and drug therapy adjustments)*’ and the corresponding main category ‘*Medication Order Review and Reconciliation*’ were addressed most frequently by the articles, with the following details provided for this clinical pharmacists’ role: A 2021 US study analyzed the results of a pharmacist-led deprescribing pilot program [36]. The number of patient encounters with the hospice pharmacist was associated with 3.2-fold higher odds of achieving more than 50% reduction in medications that were recommended for deprescribing. Another article introduced the DE-PHARM initiative, which was aimed at ensuring patient-centered, health-focused, prognosis-appropriate, and rational medication regimens [50]. DE-PHARM was a pharmacist-driven program for deprescribing, focusing on patients with limited life expectancy in long-term care. Other articles also highlighted the importance of deprescribing and discontinuing medications as pharmacy services in hospice care [33, 34, 41, 43, 47, 49].

Fourteen articles reported impact on clinical outcomes of the clinical activities (n = 14/22, 63.6%). However, only five reported measurable impacts on clinical outcomes. Five articles reported findings from cost analyses (see Table 2) with all demonstrating an association between the provision of pharmacy services and potential cost savings [26, 33, 35, 37, 51]. One of these articles showed that there was no significant association between the time spent by the pharmacist performing services and the number of medication requirements and per diem medication costs, regardless of the pharmacist chosen by the hospices (prescription benefits manager, pharmacist on staff or both) [35]. Another article demonstrated not only a favorable return on investment (based on cost avoidance due to preventable adverse drug events identified by pharmacist) that exceeds a pharmacist’s annual salary but also stated that pharmacy services ‘contribute to the quality and value of care provided to a PC patient and their family, in a way that is left unsatisfied if this discipline’s perspective is not included in the care equation’ [37].

## Discussion

### Summary of evidence

Based on our scoping review, we identified various clinical pharmacy activities and their impact on clinical outcomes that helped to gauge the scope of pharmacists’ roles in inpatient HPC. Interestingly, most publications on the topic originated from the USA. Although the modern hospice care movement was initiated in England in the nineteen-sixties [52], our search yielded only three articles from European countries. One possible explanation is that clinical pharmacy is well established in the USA, with high levels of specialized education available for pharmacists [53]. For example, US pharmacists can be trained specifically in HPC [19, 32].

The identified pharmacists’ activities and clinical roles are mainly associated with intentions to increase medication safety, and thus, patient safety. The activities involve medication counselling and education of health care providers, patients, and their families, optimization of medication regimens and therapy adaptions, as well as medication and symptom management provided in the context of direct patient care.

Due to the inherent complexity of the drug therapy regimen, at every stage of palliative care, therapy decisions and changes to the drug regimen are prone to DRPs. To avoid occurrence of DRPs, to early identify and resolve occurring DRPs, HPC settings could potentially benefit from medication reconciliation, medication review, and pharmacist-led deprescribing [33, 34, 54]. Early identification of DRPs [9, 54] paired with interprofessional communication are effective pharmacists’ activities that have been demonstrated to increase medication safety in various settings [9]. To help prevent future DRPs, alongside pharmacist-led optimization of medication regimens, drug consultations are valuable in HPC settings both to prevent, to identify, and resolve DRPs [36, 50].

Complex, frequently changing drug therapy regimens in HPC patients require thorough assessment and interprofessional exchange. A reflective approach with medications is central to a safe and rational drug regimen and particularly relevant in HPC, where patients are highly vulnerable to issues that could reduce their quality of life even for a short time [8]. Therapeutic goals change drastically with the decision to pursue non-curative treatment in favor of symptom management and quality of life. Goals must constantly be assessed and adapted, including patients’ and their families’ individual goals and needs. Thus, the adaption of medication can vary greatly over time [55]. Deprescribing is an important step to reduce polypharmacy and outweigh the benefit of each drug against its possible harm [56]. The beneficial effects of pharmacist-led deprescribing and interventions to discontinue medications were discussed in several publications [33, 36, 50]. The importance of drug therapy adaptions was reflected in the numerous articles addressing the main category ‘*Medication Order Review and Reconciliation*’*.*

The variety of pharmacists roles and clinical activities were not only shown to have a positive impact on clinical outcomes but also to be associated with lower per patient-day drug costs and cost avoidance associated with adverse drug events that were identified and resolved by pharmacists. Similar findings emerged from a 2004 survey study, where respondents reported lower overall pharmaceutical costs attributed to hiring a clinical pharmacist [57].

### Strengths and weaknesses

The MeSH term ‘Palliative Care’ is rather broad and may include publications that address earlier stages of PC (e.g., at diagnosis) rather than the last 6 months of life as defined in this scoping review. Therefore, it was not included in the search strategy. By restricting our search, we risked missing relevant publications. Definitions and concepts of HPC settings differ internationally [25], nevertheless, we expect only slight differences in the scope of pharmacists’ roles and clinical activities as complexity of drug regimens is associated with HPC in general, irrespective of the setting. The descriptive design of the scoping review has served the purpose to provide an overview of the evidence of the role of pharmacists in inpatient HPC well.

The study types revealed a high level of heterogeneity. However, this was addressed by introducing the two article categories *practice research report* and *practice report*. Further, there was a deviation in the denotation of the described clinical activities bearing a risk of mapping them to the wrong category. Standardized categorization was achieved by discussing data charting among the authors. Using the ASHP guidelines [19] as a framework was helpful to structure the findings from the literature. The structured process helped to assess the scope of pharmacists’ clinical activities and services provided to inpatient HPC. Pharmacy services are highly advanced in the US with different fields of specialization available for clinical pharmacists. Therefore, the ASHP guidelines could serve as framework to define the role of pharmacists in HPC in other countries.

Gaps in the availability of definitions of the pharmacist’s role in inpatient HPC can be used as food for thought for further research.

### Further research

Even in countries where ΗPC settings are small and the variety of prescribed medications is limited, pharmacists’ skills and knowledge can support health care providers in the medication management, especially in HPC patients with complex drug therapy regimens that are prone to DRPs. In order to develop a structured definition of the role of pharmacists in HPC, more research on clinical pharmacy activities assessing their impact on clinical outcomes and overall costs of care is needed.

## Conclusion

This scoping review identified a variety of pharmacists’ clinical roles and activities in inpatient hospice and palliative care. The most notable of the identified clinical activities were optimizing medication regimens to reduce inappropriate medication and associated risks for drug-related problems as well as the provision of medication counseling to health care providers, patients, families, and caregivers. The findings helped to highlight pharmacist contributions to inpatient hospice and palliative care. The gathered evidence will help to establish and define the role of pharmacists in inpatient hospice and palliative care settings.

## Supplementary Information

Below is the link to the electronic supplementary material.

Supplementary file 1 (PDF 93 KB)

Supplementary file 2 (PDF 59 KB)
