# Supplementary material for: Pharmacists’ clinical roles and activities in inpatient hospice and palliative care: a scoping review
Source: Int J Clin Pharm. 2023 Feb 11;45(3):577–86. doi: 10.1007/s11096-023-01535-7 (PMC9918816; doi:10.1007/s11096-023-01535-7)
Supplement: Supplementary file 1 — (PDF 93 KB) [file 11096_2023_1535_MOESM1_ESM.pdf]

S1: Search strings of each database

Topics	Database	MeSh-Terms	Keywords	Search string	Results
Pharmaceutical Services & Hospice & Pharmacist	Medline	pharmaceutical services[Mesh:NoExp]<br>medication therapy management[MeSH]<br>patient safety[MeSH]<br>polypharmacy[MeSH]<br>medication reconciliation[MeSH]<br>deprescriptions[MeSH]<br>inappropriate prescribing[MeSH]<br>pain management[MeSH]<br>potentially inappropriate medication list [MeSH]<br><br>hospices [MeSH]<br>hospice care[MeSH]<br>terminal care [Mesh:NoExp]<br>terminally ill[MeSH]<br>palliative medicine[MeSH]<br><br>Pharmacists[Mesh]	pharmac* service*[tiab]<br>medication therapy management[tiab]<br>drug therapy management[tiab]<br>pharmaceutical car*[tiab]<br>patient safety[tiab]<br>medication reconciliation[tiab]<br>drug reconciliation[tiab]<br>deprescri*<br>inappropriate prescri*[tiab]<br>potentially inappropriate medication list[tiab]<br>polypharmacy [tiab]<br>medication review[tiab]<br>drug review[tiab]<br>medication analys*[tiab]<br>drug analys*[tiab]<br>pain management[tiab]<br>symptom management[tiab]<br><br>hospice*[tiab]<br>terminal car*[tiab] terminally ill[tiab]<br>end of life care*[tiab]<br>EOL care[tiab]<br>EOLC[tiab]<br>palliative[tiab]<br><br>pharmacist*[tiab]	(((pharmaceutical services[Mesh:NoExp] OR medication therapy management[MeSH Terms] OR patient safety[MeSH Terms] OR polypharmacy[MeSH Terms] OR medication reconciliation[MeSH Terms] OR deprescriptions[MeSH Terms] OR inappropriate prescribing[MeSH Terms] OR pain management[MeSH Terms] OR potentially inappropriate medication list [MeSH Terms]) OR (pharmac* service*[tiab] OR medication therapy management[tiab] OR drug therapy management[tiab] OR pharmaceutical car*[tiab] OR patient safety[tiab] OR medication reconciliation[tiab] OR drug reconciliation[tiab] OR deprescri* OR inappropriate prescri*[tiab] OR potentially inappropriate medication list[tiab] OR polypharmacy [tiab] OR medication review[tiab] OR drug review[tiab] OR medication analys*[tiab] OR drug analys*[tiab] OR pain management[tiab] OR symptom management[tiab])) AND ((hospices [MeSH Terms] OR hospice care[MeSH Terms] OR terminal care[Mesh:NoExp] OR terminally ill[MeSH Terms] OR palliative medicine [MeSH Terms]) OR (hospice*[tiab] OR terminal car*[tiab] OR terminally ill[tiab] OR end of life care*[tiab] OR EOL care[tiab] OR EOLC[tiab] OR palliative[tiab])) AND ((Pharmacists[Mesh]) OR (pharmacist*[tiab])))	February 10th, 2021<br>→ 227

Topics	Database	MeSh-Terms	Keywords	Search string	Results
Pharmaceutical Services & Hospice & Pharmacist	Embase	'pharmaceutical services'/de 'medication therapy management'/exp 'patient safety'/exp 'polypharmacy'/exp 'medication reconciliation'/exp 'deprescriptions'/exp 'inappropriate prescribing'/exp 'pain management'/exp 'potentially inappropriate medication list'/exp<br><br>'hospices'/exp 'hospice care'/exp 'terminal care'/de 'terminally ill'/exp 'palliative medicine'/exp<br><br>'pharmacists'/exp	'pharmac* service*':ti,ab<br>'medication therapy management':ti,ab<br>'drug therapy management':ti,ab<br>'pharmaceutical car*':ti,ab<br>'patient safety':ti,ab<br>'medication reconciliation':ti,ab<br>'drug reconciliation':ti,ab<br>deprescri*<br>'inappropriate prescri*':ti,ab<br>'potentially inappropriate medication list':ti,ab<br>polypharmacy:ti,ab<br>'medication review':ti,ab 'drug review':ti,ab<br>'medication analys*':ti,ab 'drug analys*':ti,ab<br>'pain management':ti,ab<br>'symptom management':ti,ab<br><br>hospice*:ti,ab<br>'terminal car*':ti,ab<br>'terminally ill':ti,ab<br>'end of life care*':ti,ab<br>'eol care':ti,ab<br>eolc:ti,ab<br>palliative:ti,ab<br><br>pharmacist*:ti,ab	('pharmaceutical services'/de OR 'medication therapy management'/exp OR 'patient safety'/exp OR 'polypharmacy'/exp OR 'medication reconciliation'/exp OR 'deprescriptions'/exp OR 'inappropriate prescribing'/exp OR 'pain management'/exp OR 'potentially inappropriate medication list'/exp OR 'pharmac* service*':ti,ab OR 'medication therapy management':ti,ab OR 'drug therapy management':ti,ab OR 'pharmaceutical car*':ti,ab OR 'patient safety':ti,ab OR 'medication reconciliation':ti,ab OR 'drug reconciliation':ti,ab OR deprescri* OR 'inappropriate prescri*':ti,ab OR 'potentially inappropriate medication list':ti,ab OR polypharmacy:ti,ab OR 'medication review':ti,ab OR 'drug review':ti,ab OR 'medication analys*':ti,ab OR 'drug analys*':ti,ab OR 'pain management':ti,ab OR 'symptom management':ti,ab) AND ('hospices'/exp OR 'hospice care'/exp OR 'terminal care'/de OR 'terminally ill'/exp OR 'palliative medicine'/exp OR hospice*:ti,ab OR 'terminal car*':ti,ab OR 'terminally ill':ti,ab OR 'end of life care*':ti,ab OR 'eol care':ti,ab OR eolc:ti,ab OR palliative:ti,ab) AND ('pharmacists'/exp OR pharmacist*:ti,ab)	February 10th, 2021<br>→ 631<br><br>Not Medline (Pubmed):<br>→ 395

Topics	Database	MeSh-Terms	Keywords	Search string	Results
Pharmaceutical Services & Hospice & Pharmacist	CINAHL	(MH "pharmaceutical services")<br>(MH "medication therapy management+") (MH "patient safety+")<br>(MH "polypharmacy+")<br>(MH "medication reconciliation+") (MH "deprescriptions+")<br>(MH "inappropriate prescribing+")<br>(MH "pain management+") (MH "potentially inappropriate medication list +")<br><br>(MH "hospices +")<br>(MH "hospice care+")<br>(MH "terminal care")<br>(MH "terminally ill+")<br>(MH "palliative medicine +"))<br><br>(MH "Pharmacists+")	(TI "pharmac* service*" OR AB "pharmac* service*")<br>(TI "medication therapy management" OR AB "medication therapy management")<br>(TI "drug therapy management" OR AB "drug therapy management")<br>(TI "pharmaceutical car*" OR AB "pharmaceutical car*")<br>(TI "patient safety" OR AB "patient safety")<br>(TI "medication reconciliation" OR AB "medication reconciliation") (TI "drug reconciliation" OR AB "drug reconciliation") deprescri*<br>(TI "inappropriate prescri*" OR AB "inappropriate prescri*")<br>(TI "potentially inappropriate medication list" OR AB "potentially inappropriate medication list")<br>(TI polypharmacy OR AB polypharmacy)<br>(TI "medication review" OR AB "medication review")<br>(TI "drug review" OR AB "drug review")<br>(TI "medication analys*" OR AB "medication analys*")<br>(TI "drug analys*" OR AB "drug analys*")<br>(TI "pain management" OR AB "pain management")<br>(TI "symptom management" OR AB "symptom management")<br><br>(TI hospice* OR AB hospice*) (TI "terminal car*" OR AB "terminal car*")<br>(TI "terminally ill" OR AB "terminally ill")<br>(TI "end of life care*" OR AB "end of life care*")<br>(TI "EOL care" OR AB "EOL care")<br>(TI EOLC OR AB EOLC) OR (TI palliative OR AB palliative)<br><br>(TI pharmacist* OR AB pharmacist*)	((((MH "pharmaceutical services") OR (MH "medication therapy management+") OR (MH "patient safety+") OR (MH "polypharmacy+") OR (MH "medication reconciliation+") OR (MH "deprescriptions+") OR (MH "inappropriate prescribing+") OR (MH "pain management+") OR (MH "potentially inappropriate medication list +")) OR ((TI "pharmac* service*" OR AB "pharmac* service*") OR (TI "medication therapy management" OR AB "medication therapy management") OR (TI "drug therapy management" OR AB "drug therapy management") OR (TI "pharmaceutical car*" OR AB "pharmaceutical car*") OR (TI "patient safety" OR AB "patient safety") OR (TI "medication reconciliation" OR AB "medication reconciliation") OR (TI "drug reconciliation" OR AB "drug reconciliation") OR deprescri* OR (TI "inappropriate prescri*" OR AB "inappropriate prescri*") OR (TI "potentially inappropriate medication list" OR AB "potentially inappropriate medication list") OR (TI polypharmacy OR AB polypharmacy) OR (TI "medication review" OR AB "medication review") OR (TI "drug review" OR AB "drug review") OR (TI "medication analys*" OR AB "medication analys*") OR (TI "drug analys*" OR AB "drug analys*") OR (TI "pain management" OR AB "pain management") OR (TI "symptom management" OR AB "symptom management"))) AND (((MH "hospices +") OR (MH "hospice care+") OR (MH "terminal care") OR (MH "terminally ill+") OR (MH "palliative medicine +")) OR ((TI hospice* OR AB hospice*) OR (TI "terminal car*" OR AB "terminal car*") OR (TI "terminally ill" OR AB "terminally ill") OR (TI "end of life care*" OR AB "end of life care*") OR (TI "EOL care" OR AB "EOL care") OR (TI EOLC OR AB EOLC) OR (TI palliative OR AB palliative))) AND (((MH "Pharmacists+")) OR ((TI pharmacist* OR AB pharmacist*))))	February 10th, 2021<br>→ 120
